# The alpha-fetoprotein serum is still reliable as a biomarker for the surveillance of hepatocellular carcinoma in Indonesia

**DOI:** 10.1186/s12876-020-01365-1

**Published:** 2020-07-09

**Authors:** Chyntia Olivia Maurine Jasirwan, Alessa Fahira, Lianda Siregar, Imelda Loho

**Affiliations:** 1 Hepatobiliary Division, Department of Internal Medicine, Faculty of Medicine, Universitas Indonesia, Cipto Mangunkusumo National General Hospital, Jakarta, Indonesia; 2 Faculty of Medicine, Universitas Indonesia, Cipto Mangunkusumo National General Hospital, Jakarta, Indonesia; 3 Division of Gastroenterology and Hepatology, Department of Internal Medicine, Faculty of Medicine, Universitas Indonesia, Dharmais National Cancer Hospital, Jakarta, Indonesia

**Keywords:** α-Fetoprotein, Hepatocellular carcinoma, Surveillance, Biomarker

## Abstract

**Background and aims:**

Hepatocellular carcinoma (HCC), the most common type of liver cancer, is one of the leading causes of cancer-related death worldwide with an inferior prognosis. In Indonesia, the average life expectancy is less than 5 months, with most patients being in an advanced stage wherein the survival rate is very low. Early detection through surveillance program is very crucial. HCC guidelines worldwide have provided surveillance recommendation through the examination of α-fetoprotein (AFP) and ultrasound for patients at risk in developing HCC. However, there have been some controversies regarding the usage of AFP concerning its low sensitivity and specificity in detecting HCC. Therefore, the effectiveness of AFP in the surveillance of HCC patients and identifying the parameters most associated with the increase of AFP ≥ 10 ng/ml in Indonesia should be evaluated.

**Methods:**

We analyzed medical records of HCC patients and those at high risk of developing HCC through cross-sectional study, including patients with cirrhosis and hepatitis B and C, from 2015 to 2017 who underwent treatment at the Cipto Mangunkusumo National General Hospital and Dharmais National Cancer Hospital, Indonesia.

**Results:**

The sensitivity and specificity of AFP in the surveillance of HCC in Indonesia with a cut-off of 10 ng/ml were 82.6 and 71.2%, respectively. The parameters most associated with the increase of AFP ≥10 ng/ml according to multivariate analysis were the etiology of hepatitis B, the stage of Barcelona Clinic Liver Cancer (BCLC) B and C, and the presence of cirrhosis, respectively.

**Conclusion:**

AFP can still be used in the surveillance of HCC in Indonesia for its high sensitivity value.

## Background

Liver cancer is known to be a significant cause of cancer-death worldwide—owing to the death of 800.000 patients each year [1]. Ninety percent of all liver cancer cases in the world are in the form of hepatocellular carcinoma (HCC), making it the most common form of liver cancer [2]. HCC is a significant health problem worldwide because of the constant risk of its known etiologies, including hepatitis B (especially in developing countries), hepatitis C, and the alarming increase of metabolic syndrome cases such as obesity and high alcohol consumption—both of which correlate with the increasing incidence of fatty liver disease.

Furthermore, HCC is regarded as deadly due to its poor prognosis. In Indonesia, the median for the survival of HCC patients in the period from 1998 to 1999 was 138 days, and in the period from 2013 to 2014 was 146 [3]—showing that patients would, in average, only have less than 5 months life expectancy which also shows a very low improvement of survival despite being 15 years apart. This happens because of the patient’s tendency to seek treatment only reaching the advanced stage, wherein the survival of patients with HCC is very low. Thus, early detection through HCC surveillance program is believed to be an indisputable strategy to prevent the patient is coming at an advanced stage [4].

A surveillance method that has been widely recommended in several guidelines globally utilizes α-fetoprotein (AFP) biomarker and abdominal ultrasonography [5, 6]. In the Cipto Mangunkusumo National General Hospital (RSCM), the surveillance of HCC is routinely held every 6 months for patients at risk of developing HCC. Surveillance of HCC is conducted by examining the liver using USG and measuring AFP in blood. The cut-off used in Indonesia and many other countries is 10 ng/ml or if there is an elevation of AFP levels compared with the previous examination. Patients who are suspected of having HCC are further diagnosed [7]. However, in recent years, there have been studies showing controversies regarding the use of AFP in clinical settings—because of the low sensitivity and specificity of AFP when used to screen HCC [8]. It has been stated in some studies that patients with HCC may not experience any elevation of AFP at all. Conversely, patients who were not diagnosed with HCC but were diagnosed with cirrhosis, cholangiocarcinoma or other tumours were found to have elevated AFP [9–12]. Recommendation regarding the use of AFP for HCC screening was thus excluded from the American Association for the Study of Liver Diseases guideline in the year 2010 [13]. AFP was also regarded as neither being sensitive nor specific for use as a diagnostic tool in the guideline published by European Association for the Study of the Liver in the year of 2012 [14]. Nevertheless, some countries in Asia still recommend the use of AFP along with USG for the screening of HCC in the guideline published by Asian Pacific Association for the Study of the Liver in the year 2010 [15], and the guideline published by China (2011) [16] and Japan (2013) [17].

There have been no previous studies that evaluate the use of AFP in the surveillance of HCC in the Indonesian population. Therefore, this study tried to assess the sensitivity and specificity of AFP in Indonesia, where the population includes patients who undergo treatment in the Cipto Mangunkusumo National General Hospital and Dharmais National Cancer Hospital, Indonesia.

## Methods

### Study design and population

This cross-sectional study was conducted using the HCC-based registry from 2015 to 2017. HCC registry of Indonesia is a database developed by the Indonesian Liver Cancer Study Group and Bayer Group which meant to record all HCC patient’s medical data in Indonesia. Currently, two centers have been enrolled in this registry of which include RSCM and RSKD. Other registries (i.e. hepatitis and cirrhosis registry of Indonesia) have been developed earlier. Enroll criteria are all patients diagnosed with HCC in both RSCM and RSKD. Data are collected by clinicians who provided care to the patient and are processed by the research assistant of the Hepatobiliary Division. All RSCM and RSKD patients high-risk of HCC (which include having hepatitis B, hepatitis C and/or cirrhosis), which are not identified as having HCC, are enrolled either in the hepatitis registry and cirrhosis registry. The diagnosis of HCC in RSCM and Dharmais National Cancer Hospital, Jakarta (RSKD), was made from patients with positive surveillance results of either elevated AFP ≥10 ng/ml or positive ultrasound, which are then confirmed by a single examination of triple-phase abdominal CT scan. If nodules were found but are not typical for HCC, we will then proceed with Primovist-enhanced magnetic resonance imaging (MRI). In some cases, HCC was confirmed by histological examination. Etiologies of HCC included in this study were separated into viral (hepatitis B and hepatitis C, or both) and non-viral (non-hepatitis B and C). Hepatitis B and C serology markers were examined. Hepatitis B was detected through the presence of hepatitis B surface antigen and hepatitis B virus (HBV) DNA, while hepatitis C was detected through the presence of hepatitis C virus (HCV) antibody and HCV RNA. The presence of cirrhosis in patients was evaluated through clinical symptoms, abdominal ultrasonography, and transient elastography.

### Data collection

**Fig. 1:**
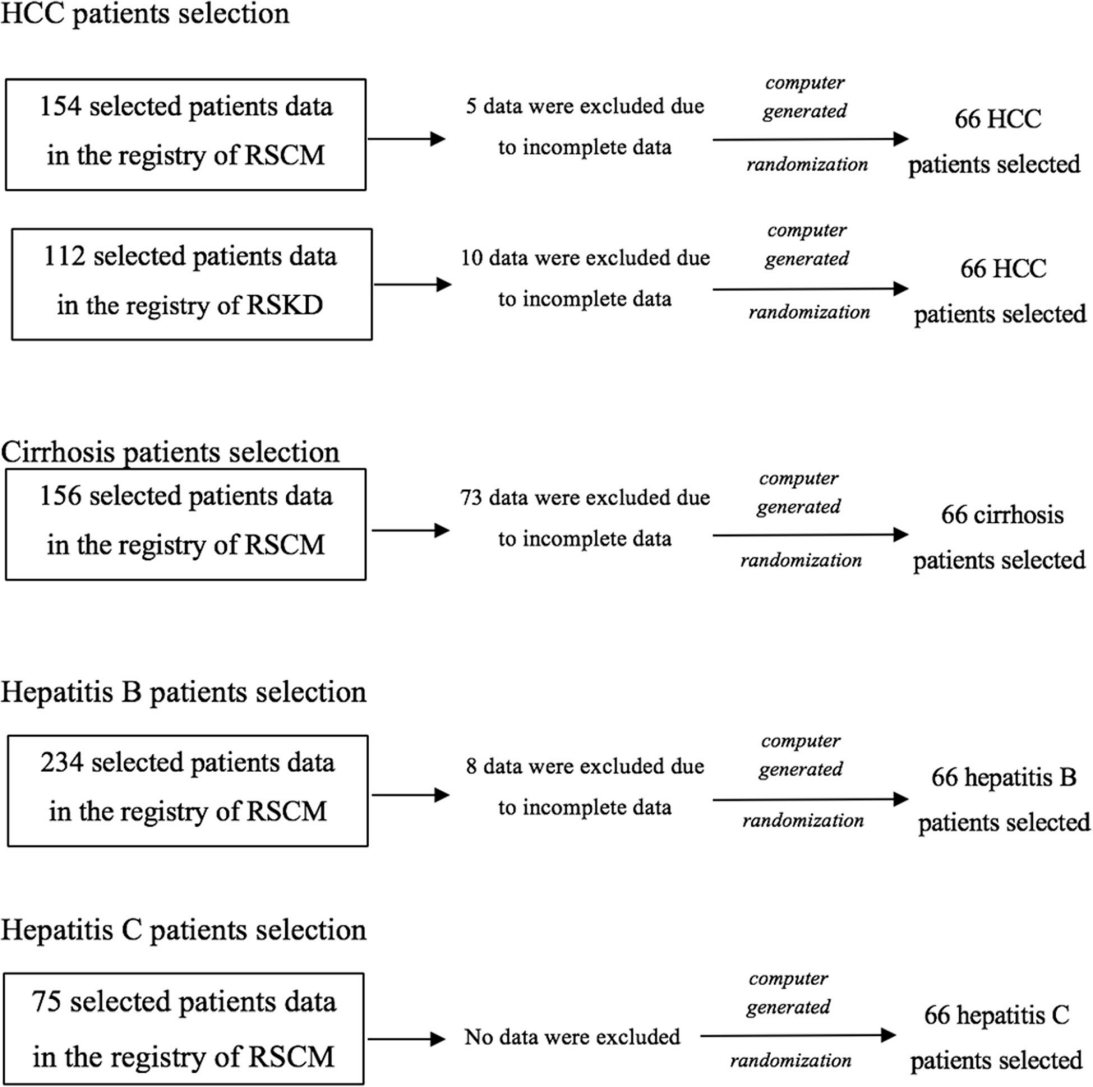
The selection process of subjects in each category. Subjects were first selected by excluding duplicates (not shown in the figure) and continued with the exclusion of incomplete data

All patients with HCC diagnosed in RSCM and RSKD between July 2015 and June 2017, whose data were recorded in the HCC registry, were analyzed (Fig. 1). Patients included in this study were HCC patients (*n* = 132) and high-risk populations including patients with cirrhosis (*n* = 66), hepatitis B patients with the absence of cirrhosis (*n* = 66), and hepatitis C patients with the absence of cirrhosis (*n* = 66). Patients with other malignancies were excluded from this study. This research was conducted in the Hepatobiliary Division, Cipto Mangunkusumo National General Hospital. Subjects were selected by computer-based randomized sampling using the SPSS application from the existing registry. The independent variable of this study was the AFP level below and above the cut-off of 10 ng/ml with 10 dependent variables including age, sex, etiology of HCC, number of nodules, nodule size, cirrhosis, portal venous thrombus, metastasis, Child-Pugh score and the staging of BCLC.

### Statistical analysis

Data processing was carried out using the IBM Statistical Package for the Social Sciences software version 21 for Mac, which included the (1) calculation of the AFP tests and gold standards to obtain sensitivity and specificity and (2) exploration of factors that might affect AFP levels in HCC patients through bivariate and multivariate analysis. The categorical variables were compared by using the chi-square test, Fisher’s test or Mann–Whitney test, if appropriate. Variables with a value of *P* ≤ 0.25 in bivariate analysis were included in multivariate logistic regression analysis.

### Ethics approval

This study was approved by the ethics committee of The Faculty of Medicine, University of Indonesia with the letter number of 0514/UN2.F1/ETIK/2018.

## Results

**Fig. 2:**
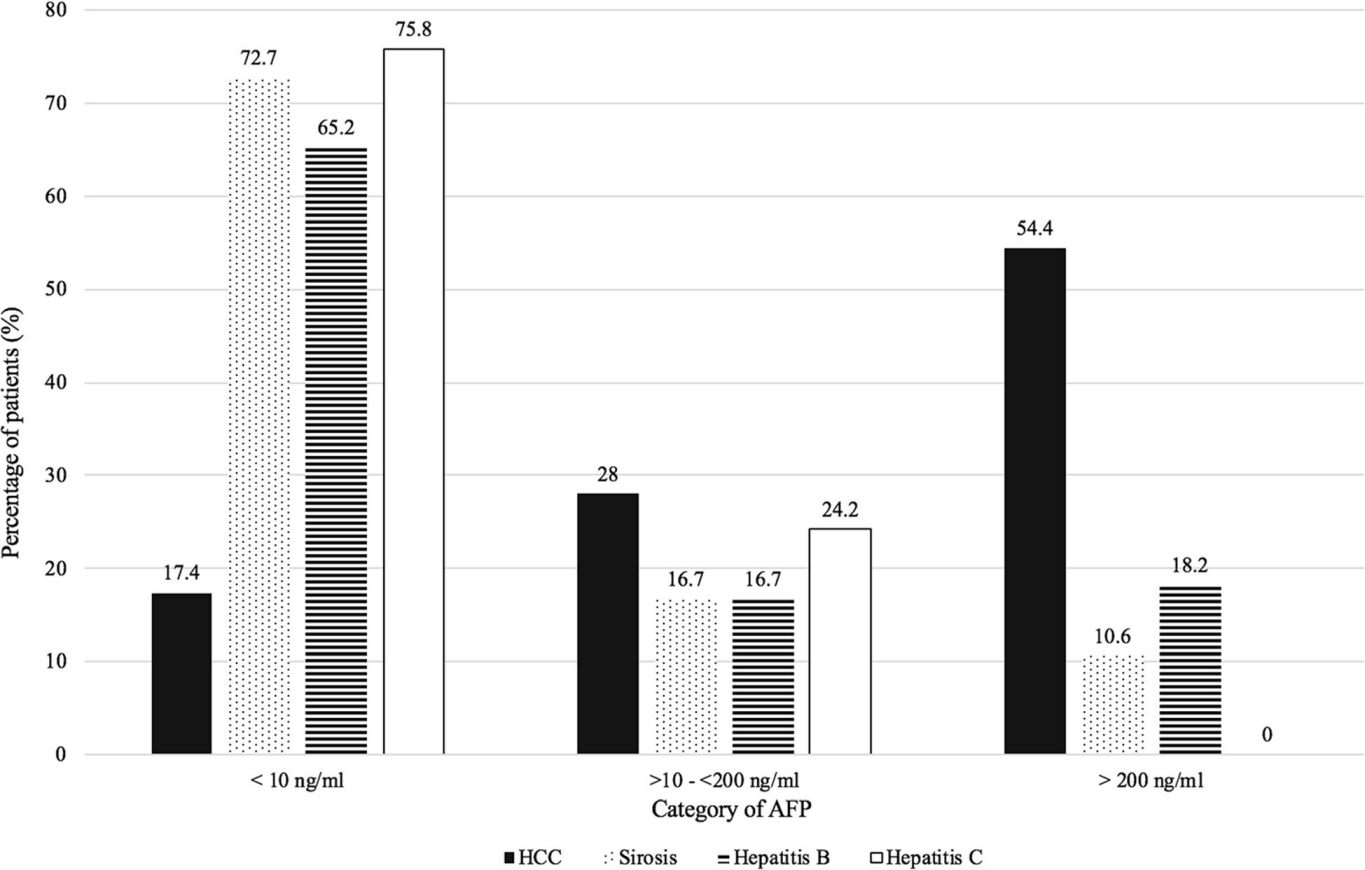
AFP distribution in HCC patients and controls. HCC patients are portrayed with the colour of black and controls (population at risk in developing HCC) which include cirrhosis, hepatitis B and hepatitis C are depicted with dots, horizontal lines and solid white, respectively

This study included 132 HCC patients and 198 patients at risk in developing HCC (66 cirrhotic patients, 66 non-cirrhotic hepatitis B patients, and 66 non-cirrhotic hepatitis C patients). The characteristic of HCC patients is portrayed in Table 1 and the AFP distribution in HCC patients and control is shown in Fig. 2. AFP levels above 10 ng/ml were seen in 82.6% (109 of 132 patients) HCC patients and 29% (57/198) non-HCC patients. In this study, all HCC patients with elevated AFP ≥10 ng/ml were found to have positive nodule (either single or multiple nodules) on ultrasound. However, 23 (17%) HCC patients with positive nodule on ultrasound were found to have normal AFP. The result of this study shown the sensitivity levels of 82.6%, specificity levels of 71.2%, the positive predictive value of 65.6%, the negative predictive value of 85.9% and the positive likelihood ratio of 2.87. False positive from all patients with AFP above 10 ng/ml was 34% (57/ 166) (Table 2).

**Table 1:** Hepatocellular carcinoma patient’s characteristic

Variable	No. of Patients (%)
Age	
< 40 years old (*n* = 12)	9.1%
40–< 50 years old (*n* = 71)	53.8%
≥ 50 years old (*n* = 49)	37.1%
Sex	
Male (*n* = 94)	71.2%
Female (*n* = 38)	28.8%
Etiology of HCC	
Hepatitis B (*n* = 84)	63.6%
Hepatitis C (*n* = 22)	16.7%
Non-Hepatitis B and Non-C (*n* = 18)	13.6%
Hepatitis B and Hepatitis C (*n* = 8)	6.1%
Cirrhosis	
No (*n* = 59)	44.7%
Yes (*n* = 73)	55.3%
BCLC staging	
A (*n* = 14)	10.6%
B (*n* = 56)	42.4%
C (*n* = 52)	39.4%
D (*n* = 10)	7.6%
Child–Pugh	
A (*n* = 81)	61.4%
B (*n* = 38)	28.8%
C (*n* = 13)	9.8%
Number of nodules	
Singular (*n* = 69)	52.3%
Multiple (*n* = 62)	47%
Diffuse (*n* = 1)	0.7%
Largest nodule size	
< 20 mm (*n* = 7)	5.3%
20–< 50 mm (*n* = 15)	11.4%
50–< 100 mm (*n* = 39)	29.5%
≥ 100 mm (*n* = 71)	53.8%
Metastasis	
No (*n* = 115)	87.1%
Yes (*n* = 17)	12.9%
Portal vein thrombosis	
No (*n* = 90)	68.2%
Yes (*n* = 42)	31.8%

**Table 2:** Sensitivity and specificity table of AFP test in the surveillance of HCC

AFP	HCC (+)	HCC (−)
AFP ≥ 10 ng/ml	109 *True Positive* (TP)	57 *False Positive* (FP)
AFP < 10 ng/ml	23 *False Negative* (FN)	141 *True Negative* (TN)

Bivariate analysis, shown in Table 3, revealed a significant relationship between AFP levels above or below 10 ng/ml and the etiology of HCC (*p =* 0.011) and cirrhosis (*p* = 0.016), yet we found no significant association with other variables. Multivariate analysis, portrayed in Table 4, revealed the parameter most associated with the risk of having an AFP level above 10 ng/ml was patients within the stage C of BCLC (OR = 16; *p =* 0.002), followed by patients the HCC etiology of hepatitis B (OR = 6.35; *p =* 0.005), cirrhosis (OR = 4.31; *p =* 0.016), and within the stage B of BCLC (OR = 5.99; *p =* 0.019), respectively.

**Table 3:** Bivariate analysis

Variables	Serum AFP: Number of Patients (%)		P
	< 10 ng/ml	≥ 10 ng/ml	
**Etiology**			
Hepatitis B	10	74	**0.011**
Hepatitis C	3	19	
Non-Hep B and C	8	10	
Hepatitis B and C	2	6	
Age (years)			
< 40	3	9	0.7
40- < 50	11	60	
≥ 50	9	40	
Sex			
Female	9	29	0.34
Male	14	80	
Size of nodule			
< 20 mm	3	4	0.401
20 - < 50 mm	2	13	
50 - < 100 mm	7	32	
≥ 100	11	60	
Nodules			
Singular	15	54	0.166
Multiple	8	54	
Diffuse	0	1	
**Cirrhosis**			
None	16	43	**0.016**
Yes	7	66	
Child-Pugh			
Class A	17	64	0.36
Class B	4	34	
Class C	2	11	
Thrombus			
None	19	71	0.165
Yes	4	38	
Metastasis			
None	20	95	0.97
Yes	3	14	
BCLC			
A	5	9	0.098
B	11	45	
C	4	48	
D	3	7

**Table 4:** Multivariate Analysis

			95% CI	
	*p*	OR	Lower	Upper
BCLC C	.002	16.024	1.309	14.236
Etiology of hepatitis B	.005	6.350	1.334	26.900
Cirrhosis	.016	4.317	2.821	91.009
BCLC B	.019	5.991	1.753	23.005

## Discussion

The population of patients at risk of developing HCC will undergo surveillance through the measurement of AFP levels and evaluation of the liver by ultrasound every 6 months. The population at risk includes patients with liver cirrhosis of any etiology and hepatitis B patients. In the Indonesian National Consensus of the Management of Hepatocellular Carcinoma [7] population at risk in developing HCC also includes chronic hepatitis C patients who developed fibrosis, but this population of patients has not yet been included in the current surveillance program in clinical practice. The cut-off of 10 ng/ml in the surveillance of HCC is deemed most appropriate as it yields high sensitivity. Although cut-off of 16–20 ng/ml resulted in higher specificity of 90%, the sensitivity would only reach 60%—hence 40% of HCC cases would be missed [18]. On the other hand, the cut-off of 10 ng/ml would reach a higher sensitivity. According to Chan SL, et al. [19] in their research on 805 patients with Asian ethnicity, the cut-off of 10 ng/ml would result in the sensitivity and specificity of 82.6 and 70.4%, respectively. This cut-off is preferable in the setting of surveillance as higher sensitivity is yielded much more of importance.

In our study, we found the sensitivity, specificity, positive predictive value, negative predictive value, and likelihood ratio for positive test results of AFP in the surveillance of HCC of AFP (with a cut-off of 10 ng/ml) was 82.6, 71.2, 65.6, 85.9% and 2.87, respectively. Interestingly, this result was in accordance with that of a study conducted in a population of 805 patients of Asian ethnicity by Chan SL, et al. [19], showing the AFP sensitivity and specificity values of 82.6% with a specificity of 70.4% (with a similar cut-off of 10 ng/ml), with the results of positive predictive values and negative predictive values obtained as 86.6 and 63.6%, respectively. On the other hand, research conducted by Biselli, et al. [20] in a population of HCC patients in Italy showed an AFP sensitivity with a cut-off of 10 ng/ml was 66.3% with a specificity of 80.6%. It should be of note that the study conducted by Chan SL [19] in Asian patients had higher sensitivity levels than that conducted by Biselli, et al. [20] Sensitivity of the AFP test in Asian countries, predominantly in developing countries such as in Indonesia, is believed to be higher because the prevalence of HCC with the etiology of hepatitis B tends to be higher—presumably due to the lower coverage of hepatitis B immunization in newborns [21].

We concluded that with a sensitivity of 82.6% and a specificity of 71.2%, HCC surveillance using AFP test with a cut-off of 10 ng/ml is still useful due to its high sensitivity—as sensitivity levels above 80% still appear to be adequate for screening programs. High specificity, on the other hand, is more useful in establishing a diagnosis, so the specificity of 71.2% is still considered sufficient in the HCC surveillance program. That being said, we still recommend the use of AFP test to also be carried out along with USG to reach a higher sensitivity and specificity level in HCC surveillance. However, it should be noted that adding AFP into the screening algorithm will most likely increase the need for CT and MRI scans to confirm the positivity of the AFP findings, as the number of false-positive was 34% in this study.

In our study, it was found that etiology was one of the factors that were statistically significant (*p* = 0.011) in its probability to cause AFP levels of HCC patients to rise above 10 ng/ml. It can be seen from Table 1 that the highest aetiological prevalence of HCC in the patient population in this study was hepatitis B, which was followed by hepatitis C, and this is as per the current data that shows that there are 400 million patients infected with HBV, 75% of whom are Asian [22]. This significant result is also similar to that of a study conducted by Murugavel KG, et al. [23] which explained that there was a higher proportion of AFP elevation in HCC patients with the etiology of hepatitis B compared with other viral etiologies. Similar to the study conducted by Liu C, et al. [24], this study showed an increase of AFP level in cases of HCC caused by HBV compared with non-hepatitis cases of HCC. Also, a study conducted by Hann, et al. [25] showed that an increase in serum AFP in hepatitis B is in line with an increased risk for HCC.

We found that in HCC patients with the etiology of hepatitis B, 88.1% of patients had an increased level of AFP above 10 ng/ml cut-off with an odds ratio (OR) of 5.92, which was statistically significant (*p* = 0.019). According to Li M, et al. [26], this tendency of an elevated AFP level in HBV-infected patients was due to the presence of HBV protein (HBx) which could induce AFP receptor regulation, thereby increasing AFP expression in HCC due to HBV infection. Research conducted by Zhang C, et al. [27] and Yao M, et al. [28] also showed that HBV co-transcription factors could directly bind to AFP gene promoters, hence increasing its expression.

In the population of HCC patients with hepatitis C etiology, higher levels of AFP (above cut-off) was also found (with the OR 5.067) compared with the population of non-hepatitis HCC patients, although this OR was not statistically significant. Studies show that a significant increase in AFP was less common in patients with HCC with hepatitis C etiology [29, 30]. Studies conducted in Egypt, the country known to have the highest prevalence of hepatitis C, showed that the prevalence of an increase in AFP levels above 10 ng/ml in HCC patients with etiology of hepatitis C was proven to be less frequent, occurring only in 11.6% patients [31]. These results were similar to those of research conducted in western countries with a higher prevalence of hepatitis C compared to hepatitis B, wherein AFP elevation above cut-off occurred only in 10–43% patients [32–34].

This study also showed a statistically significant difference in the proportion of HCC patients with cirrhosis and non-cirrhosis, when compared with AFP levels above and below 10 ng/ml (*p* = 0.016). There were 55.3% (*n* = 73) patients with cirrhosis in this study. We also found that HCC patients with cirrhosis would have a higher risk of having an elevated AFP level above cut-off (OR 3.508, *p* = 0.011). These results were similar to those of previous studies, as cirrhosis is one of the main known risk factors for HCC, and is found in 80–90% of all HCC cases. HCC is also one of the causes of death in patients with cirrhosis of the liver, with the rate of developing HCC in patients with liver cirrhosis per year being 5% [35, 36]. This causes all patients with cirrhosis with any etiology to be recommended through surveillance of HCC [36, 37]. Pathological study has shown that patients with chronic liver disease can express AFP without prior development of HCC [38]. The study by Harada T, et al. [39] showed that approximately 40% of all cirrhotic patients will have AFP levels higher than 20 ng/ml. An increase in AFP levels (between 10 and 500 ng/ml, and sometimes up to 1000 ng/ml) can be seen in adult patients with hepatitis or cirrhosis with any etiology. Also, the frequency of elevated AFP levels (> 10 ng/mL) was reported in 20% of cases of chronic hepatitis and 40% of cases of cirrhosis [40].

This study also found that BCLC stage C (*p* = 0.002), etiology of hepatitis B (*p* = 0.005), cirrhosis (*p* = 0.016) and BCLC B stage (*p* = 0.019) were all independent predictors of elevated AFP levels above 10 ng/ml. The BCLC C classification, which was considered an advanced stage, classified patients who had already developed tumours invading the portal vein and those who had already developed extrahepatic dissemination. The condition of the spread of these tumours, according to literature [41], was related to higher AFP levels, although this was not statistically proven in this study. However, it should be taken into account that detecting the increase AFP level in the earlier stages is paramount. In our study, there were 14 (10,6%) BCLC stage A HCC patients, whom 9 (64,2%) were found to have an increase AFP level (Table 3). This emphasizes the advantage of using AFP as a surveillance tool even within the earlier stages. However, further investigation of the diagnostic accuracy in the earlier stage would require a more adequate number of subjects and is an interesting study that should be conducted. In our setting, as most of HCC patients were already in advance stages and the included HCC population in this study was all ultrasound positive, the additive value of AFP may seem unclear. Nevertheless, Singal et al. [42] stated if ultrasound was the only method of surveillance used in detecting HCC, the sensitivity will only reach 58%, whereas when combined with AFP, the sensitivity will increase to 87%. Questions regarding the additive value of AFP if compared to ultrasound only is intriguing—and further study should be conducted.

## Conclusion

The sensitivity level of 82.6% and specificity level of 71.2% in the surveillance of HCC using AFP with a cut-off of 10 ng/ml are considered useful in Indonesia. The multivariate analysis showed the factors most associated with an increase in AFP levels above 10 ng/ml were BCLC C (*p* = 0.002), the etiology of hepatitis B (*p* = 0.005), cirrhosis (*p* = 0.016) and BCLC B (*p* = 0.019).

## Data Availability

The datasets used in the study are available from the corresponding author upon reasonable request.

## Abbreviations

HCC: Hepatocellular carcinoma
AFP: α-fetoprotein
RSCM: Cipto Mangunkusumo National General Hospital, Jakarta
RSKD: Dharmais National Cancer Hospital, Jakarta
MRI: Magnetic resonance imaging
HBV: Hepatitis B virus
HCV: Hepatitis C virus
BCLC: Barcelona Clinic Liver Cancer
OR: Odds ratio
HBx: HBV protein
